# Intention offloading: Domain-general versus task-specific confidence signals

**DOI:** 10.3758/s13421-024-01529-4

**Published:** 2024-02-21

**Authors:** Chhavi Sachdeva, Sam J. Gilbert

**Affiliations:** 1 Institute of Cognitive Neuroscience, University College London, London, UK; 2 Faculty of Psychology, Swiss Distance University Institute, UniDistance Suisse, Schinerstrasse 18, 3900 Brig, Switzerland

**Keywords:** Metacognition, Cognitive offloading, Distributed cognition, Intention offloading

## Abstract

Intention offloading refers to the use of external reminders to help remember delayed intentions (e.g., setting an alert to help you remember when you need to take your medication). Research has found that metacognitive processes influence offloading such that individual differences in confidence predict individual differences in offloading regardless of objective cognitive ability. The current study investigated the cross-domain organization of this relationship. Participants performed two perceptual discrimination tasks where objective accuracy was equalized using a staircase procedure. In a memory task, two measures of intention offloading were collected, (1) the overall likelihood of setting reminders, and (2) the bias in reminder-setting compared to the optimal strategy. It was found that perceptual confidence was associated with the first measure but not the second. It is shown that this is because individual differences in perceptual confidence capture meaningful differences in objective ability despite the staircase procedure. These findings indicate that intention offloading is influenced by both domain-general and task-specific metacognitive signals. They also show that even when task performance is equalized via staircasing, individual differences in confidence cannot be considered a pure measure of metacognitive bias.

## Introduction

People often use external reminders to help them remember delayed intentions. This is known as “intention offloading” (Gilbert et al., 2020), which is a specific example of “cognitive offloading.” Cognitive offloading is defined as the broader phenomenon of using physical action to reduce cognitive demands (Risko & Gilbert, 2016). Previous research investigating the causes of offloading (Ball et al., 2021; Gilbert, 2015a, 2015b; Gilbert et al., 2020; Sachdeva & Gilbert, 2020) has found a relationship between confidence and intention offloading behavior (Boldt & Gilbert, 2019; Gilbert, 2015a, 2015b; Gilbert et al., 2020) where individuals with lower confidence in their memory ability are more likely to set reminders, regardless of their actual memory ability. Similar links between confidence and cognitive offloading in other domains have also been reported (Dunn & Risko, 2016; Hu et al., 2019). This study aimed to extend this line of research by examining the link between confidence and intention offloading behavior. Specifically, we examined the extent to which offloading is associated with domain-general versus task-specific metacognitive signals.

### Metacognition

Metacognition refers to the ability to monitor mental processes, often for the purpose of cognitive control (Flavell, 1979). It has been studied in a wide variety of domains including decision making (e.g., Yeung & Summerfield, 2012), memory (e.g., Nelson & Narens, 1990), strategic intention offloading (e.g., Gilbert, 2015a; Gilbert et al., 2020), and visual perception (e.g., Song et al., 2011). This raises questions of whether metacognitive representations such as estimates of confidence are based on domain-general versus task-specific signals.

Research in metacognition has identified two separate measures of metacognition (Fleming & Lau, 2014). The first is referred to as *metacognitive bias*, which refers to the overall tendency of an individual to report high or low confidence regardless of their performance. The second is called *metacognitive sensitivity*, which refers to the ability of an individual to discriminate between different levels of their performance, such as correct and incorrect responses. The current study examined domain-general signals of metacognitive bias in memory and perceptual tasks. Furthermore, metacognitive sensitivity was also measured, but only in the perceptual tasks.

The domain-general view of metacognition proposes that individuals use a shared metacognitive signal when evaluating their performance across different types of task (de Gardelle & Mamassian, 2014; Faivre et al., 2017). In contrast, the domain-specific account of metacognition states that distinct metacognitive resources are leveraged when individuals evaluate their performance across different types of tasks (Morales et al., 2018). By looking at confidence correlations between domains, it is possible to differentiate between the two proposals. For example, if individuals display high confidence in one task and also show high confidence in another task of a different domain, this would lend support to the domain-general account of metacognition (see Baird et al., 2013, for a domain-general account of metacognitive bias). But this is only true if confidence is dissociated from performance (e.g., with a staircase procedure) otherwise correlated confidence might just reflect correlated task performance. If, however, there are no correlations between cross-domain tasks, this would provide support for the domain-specific account of metacognition (see Baird et al., 2013, for a domain-specific account of metacognitive sensitivity).

Understanding the domain-generality of confidence in cognitive offloading could have important implications. For example, if cognitive offloading is influenced by domain-general metacognitive signals, this would suggest that a metacognitive intervention that alters an individual’s confidence in one domain could influence cognitive offloading strategies across multiple domains. In contrast, if cognitive offloading is influenced by task-specific metacognitive signals, this would suggest the need for task-specific metacognitive interventions.

### The role of domain-general confidence in intention offloading

Gilbert (2015b, Experiment 2) first explored domain-general confidence processes in intention offloading in a web-based task. The study investigated (1) whether metacognitive bias in two perceptual tasks not only correlated across those tasks but also correlated with confidence in a memory task, and (2) whether perceptual confidence correlated with participants’ likelihood of setting reminders in a memory task (referred to in this paper as offloading proportion).

In this study, participants were presented with a memory task and a pair of perceptual discrimination tasks. On each perceptual trial, participants had to provide metacognitive evaluations of how confident they were that they responded correctly on that trial. A staircase procedure was used to stabilize performance at around 70% accuracy. Using trial-by-trial metacognitive evaluations meant that both measures of metacognition, bias (calculated as mean confidence rating across trials) and sensitivity could be derived.

Gilbert (2015b, Experiment 2) found that within the two perceptual tasks, confidence and metacognitive sensitivity were independent of each other, but they were correlated with their corresponding measure in the other task. This result supports the notion of a shared metacognitive resource between the two perceptual tasks (see de Gardelle & Mamassian, 2014). This study also found that perceptual confidence positively correlated with confidence in the memory task. This suggests a domain-general component to confidence where perceptual confidence can predict confidence in a mnemonic task even when it does not predict task performance in the perceptual tasks (as this was equalized using a staircase procedure). Moreover, Gilbert (2015b, Experiment 2) found that confidence in perceptual tasks negatively predicted the proportion of reminders participants set in the memory task. In other words, participants who displayed lower confidence ratings in the perceptual tasks also set more reminders in the memory task, which again suggests domain-general signals of confidence across the two task domains.

Metacognitive sensitivity, however, was found to be domain-specific as it correlated with its corresponding measure in the perceptual tasks but did not correlate with any other measure in the perceptual task nor with any of the measures in the memory task (Gilbert, 2015b, Experiment 2). Although there are some studies that have found a domain-general component to metacognitive sensitivity (e.g., Mazancieux et al., 2020), the results of Gilbert (2015b, Experiment 2) are consistent with the majority of studies in confidence literature that have concluded in favor of a domain-specific account for metacognitive sensitivity (Baird et al., 2013, 2015; Lee et al., 2018; McWilliams et al., 2022; Morales et al., 2018).

### Biases and optimality in intention offloading

In everyday life, it is not possible to set reminders for every intended activity, including routine everyday tasks such as eating and sleeping. Individuals need to weigh the costs of setting reminders (e.g., the time and effort of reminder-setting) against the benefits. How optimal are these decisions? While the paradigm used by Gilbert (2015b) can be used to measure a participant’s choice between remembering an intention using their own memory or using an external reminder, it cannot answer the question of which choice is optimal. This is because there is no obvious way to determine which strategy is correct in this paradigm. To address the question of optimality, Gilbert et al. (2020) developed an updated experimental paradigm in which participants had to explicitly weigh the costs and benefits of setting reminders.

In this paradigm, participants performed a difficult task in which accuracy is low (approximately 50%) when using internal memory, but close to 100% when using external reminders. Participants were given a series of choices between earning maximum points using their own memory (10 points per remembered item) or a smaller number of points (between 1 and 9) with reminders. This allowed for the examination of optimality of choice behavior. For example, if a participant can achieve 65% accuracy using internal memory and 100% accuracy using reminders, it would be optimal for them to choose internal memory when offered 6 points or below per item with reminders and external reminders when offered 7 points or above per item.

In their study, Gilbert et al. (2020) found that participants were systematically biased towards using reminders even when they could have earned more points using their own memory. This is called reminder bias. Gilbert et al. (2020) also found that individual differences in this reminder bias were stable over time and correlated with participants’ metacognitive bias where to the extent that an individual was underconfident in their memory ability, they were more likely to show a bias towards reminders.

It should be noted that reminder bias is a different measure to the offloading proportion measure used by Gilbert (2015b). While offloading proportion refers to the proportion of targets for which participants set reminders, regardless of the optimal strategy (Gilbert, 2015a, 2015b) (i.e., their *propensity* to offload), reminder bias refers to bias towards or away from using reminders compared with optimal strategy (i.e., their *preference* to offload). This reminder bias in turn depends on an individual’s level of memory performance and reflects the individual’s inclination towards or away from setting external reminders. The key difference between the preference for offloading (reminder bias) versus the propensity to offload (offloading rate) is that the former takes account of underlying memory ability, but the latter does not. A particular rate of offloading might have very different meaning for two individuals. For someone with poor memory ability, a particular offloading rate might reflect inadequate use of reminders. The same rate of offloading might reflect excessive use of reminders in a person with good memory ability. Although Gilbert (2015b, Experiment 2) looked at the relationship between perceptual confidence and offloading rate, they did not examine reminder bias.

In the current study, the concept of optimality is important as it pertains to individuals’ decisions to set external reminders. Optimality, in this context, refers to the extent to which participants make choices that maximize their overall task performance. This concept is important because it helps us understand the efficiency of individuals’ decision-making where they need to weight the costs and benefits of setting reminders. By making use of this paradigm in the current study, we aim to uncover the role of domain-general and task-specific confidence signals in influencing participants’ choices and to what extent these choices align with optimal strategies.

### Current study

The main aim of the current study was to extend the findings of Gilbert (2015b, Experiment 2) to investigate whether domain-general confidence signals are linked to individuals’ bias towards or away from setting a reminder (i.e., reminder bias).

To investigate this, the current study employed three tasks. Two of these were perceptual tasks, while the third was a memory task. As in Sachdeva and Gilbert (2020) the memory task was accompanied by two confidence judgment scales. The first one was presented just before the experimental trials (pre-task confidence judgment) and the second one was presented after the experimental trials (post-task confidence judgment). The perceptual tasks used in this experiment were the same as those in Gilbert (2015b Experiment 2). As in Gilbert (2015b Experiment 2), a staircase procedure was used to stabilize accuracy in the perceptual tasks at around 70%. Using a staircase procedure limits individual differences in task performance, which means that individual variation in confidence represents bias rather than true differences in actual task performance.

Instead of calculating metacognitive sensitivity with the AUROC2 measure used in Gilbert (2015b Experiment 2), the current study quantified metacognitive sensitivity as metacognitive efficiency using the *M*-ratio (meta-*d’*/*d’*) (Maniscalco & Lau, 2012). The reason for using the *M*-ratio was because research has suggested that measures used to quantify type 2 sensitivity (i.e., the ability to distinguish correct from incorrect responses, such as the AUROC2) do not control for the effect of type 1 sensitivity (i.e., first-order task performance) (Fleming & Lau, 2014). This means that spurious correlations in metacognitive sensitivity might emerge between domains that are driven by variation in task performance rather than metacognitive capacity itself (Rouault et al., 2019).

The *M*-ratio attempts to control for this variation in task performance. The *meta-d’* framework models the relationship between performance and metacognition where *meta- d’* is defined as the first-order task performance (*d’*) that would lead to the observed type II ROC curve in the absence of noise or imprecision in confidence estimates (Maniscalco & Lau, 2012). *Meta-d’* quantifies the sensitivity of confidence ratings to performance in units of *d’*, which is the signal available for a participant to perform the type II task (Maniscalco & Lau, 2012). As *d’* and *meta-d’* are quantified in the same units, they can be compared with each other while controlling for task performance.

The findings of this research will contribute to the broader understanding of metacognition and cognitive processes by investigating the interplay between domain-general and domain-specific metacognitive signals on performance in a mnemonic task.

### Hypotheses

The key hypotheses for our study were as follows:

- We predicted a positive correlation between participants’ confidence in the perceptual and memory tasks, where participants who reported better performance in the perceptual discrimination tasks would also predict better performance in the memory task.
- We hypothesized that reminder bias would have a negative correlation with memory confidence, where participants who have lower confidence in the memory task would also be more biased towards setting reminders.
- We predicted a negative correlation between participants’ confidence in the perceptual tasks and reminder bias in the memory task, where participants who display lower confidence in the perceptual tasks would also be more biased towards setting reminders in the memory task.
- We hypothesized that metacognitive sensitivity would be domain-specific, where it would correlate with its corresponding measure across the two perceptual tasks but would not correlate with any measure in the memory task.

Before commencing data collection, the hypotheses, exclusion criteria, experimental procedure, and data analysis plan were pre-registered (https://osf.io/9efjb/).

## Method

### Participants

A total of 138 participants (88 male; 50 female; mean age: 25.9 years; SD age: 7.7 years; range: 18–55 years) were recruited from Prolific (https://www.prolific.co), an online platform in which participants receive payment for their completion of web tasks. Participation was restricted to volunteers aged 18 years or above who were English speaking and resided in either the UK or the USA. Ethics approval for this study was received by UCL Research Ethics Committee (1584/003).

To determine sample size, a statistical power analysis was performed using G*Power 3.1 (Faul et al., 2007). We wanted to power our study to detect an effect where the reminder bias in the offloading task could be predicted by metacognitive confidence in the perceptual discrimination tasks. To calculate this predicted effect size, we used the results of Kirk et al. (2021), who found a significant correlation between metamemory bias and reminder bias (*r* = -.34). Gilbert (2015b, Experiment 2) found that offloading was correlated with both perceptual metacognition and metamemory. However, the correlation between perceptual metacognition and the offloading measure was weaker (*r* = -.13) than the correlation between metamemory and the offloading measure (*r* = -.21). Therefore, we used the proportional reduction in strength between perceptual metacognition and metamemory from the Gilbert (2015b, Experiment 2) study (38%), and applied this reduction to the correlation found by Kirk et al. (2021) (*r* = -.34). This yielded a new *r* of .21. To achieve 80% power to obtain an effect of this size (one-tailed test, *α* = .05), an estimated sample size of 138 participants would be required.

If participants were excluded (*n* = 11) based on our pre-registered criteria (see below), additional participants were recruited to ensure a final sample of 138 participants. Participation in the study took approximately 60 min, and participants were compensated £7.50 for their time.

### Design

The task was programmed in Java using Google Web Toolkit version 2.8 (http://www.gwtproject.org) and Lienzo graphics toolbox version 2.0 (http://emitrom.com/lienzo), implemented in Eclipse (https://www.eclipse.org).

The paradigms used by Gilbert (2015b, Experiment 2) and Gilbert et al. (2020, Experiments 2 and 3) were adapted to investigate whether confidence can be generalized across memory and perceptual tasks. All participants completed three tasks in this experiment. One of these tasks was a memory task (see Gilbert et al., 2020; Kirk et al., 2021; Sachdeva & Gilbert, 2020) and the other two were perceptual discrimination tasks (see Gilbert, 2015b, Experiment 2). The order of the tasks was counterbalanced so that participants were randomly allocated to perform the memory task before the perceptual discrimination tasks or vice versa.

#### Perceptual tasks

**Fig. 1:**
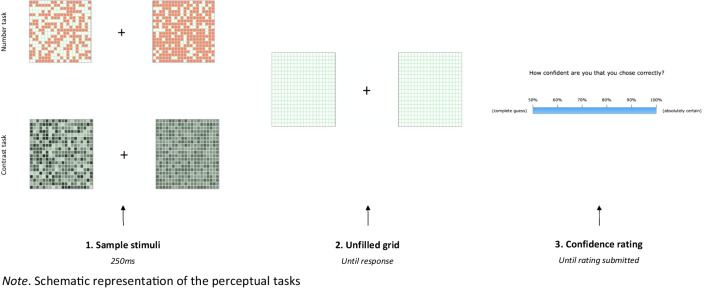
Participants complete two perceptual tasks, a Number task and a Contrast task. They then submit their response where they either judge which side contained more filled squares (Number task), or decide which side had a greater contrast between the different shades. Finally, the submit their confidence rating

Like Gilbert (2015b, Experiment 2), the current study had two perceptual tasks, a Number task and a Contrast task (see Fig. 1). Accuracy on the perceptual tasks was maintained at about 70% using a two-down-one-up staircase procedure (as in Gilbert, 2015b, Experiment 2), where difficulty increased by one step after two consecutive correct responses and decreased by one step if any incorrect responses were made.

In both perceptual tasks participants viewed a fixation point with a pair of grids positioned on either side of it. Both grids were made up of 20 horizontal and 20 vertical pale green lines to yield a total of 400 internal squares. In the Number task, 200 random squares on one side were filled in pink and on the other side more than 200 random squares were filled in pink. Participants were asked to judge which side contained more filled squares. Starting difficulty of this task was set so that 300 squares were filled on one side. To increase the difficulty of the task, this number was gradually reduced so that the side with more squares started to approach 200. Participants received two blocks of 75 main experimental trials of this task. A demonstration of the Number task can be accessed at: https://cognitiveoffloading.net/chhavi/numberDemo/WebTasks.html.

In the Contrast task, the squares within each grid were filled with a different shade of grey and participants were asked to judge which grid had a greater contrast between the different shades. Initial difficulty of this task was set so that the shades on one side varied from 15% maximum brightness to 85%, and the shades on the other side varied between 35% and 65%. To increase the difficulty of this task, the difference between the two sides was gradually reduced to approach 25–75% on both sides. Brightness of the 400 squares was uniformly distributed from brightest to darkest. Participants received two blocks of 75 main experimental trials of this task. A demonstration of the Contrast task can be accessed at: https://cognitiveoffloading.net/chhavi/contrastDemo/WebTasks.html.

On each perceptual trial, participants had to discriminate between two perceptual stimuli. After responding, they were asked to give a rating of how confident they were that they responded correctly on that trial. They could make a response by clicking anywhere on a clickable continuous scale. This scale ranged from 50% (their response was a complete guess) to 100% (absolutely certain that they responded correctly). In addition to these trial- by-trial confidence responses, they were also asked to provide an overall post-task confidence rating after completing each perceptual task (see Gilbert, 2015b, Experiment 2). This rating was given using a moveable slider ranging from 50% (they thought that every response was a complete guess) to a 100% (they thought that they got every single perceptual discrimination correct) given that chance level for a two-alternative forced-choice (2AFC) task is 50%.

#### Memory task

**Fig. 2:**
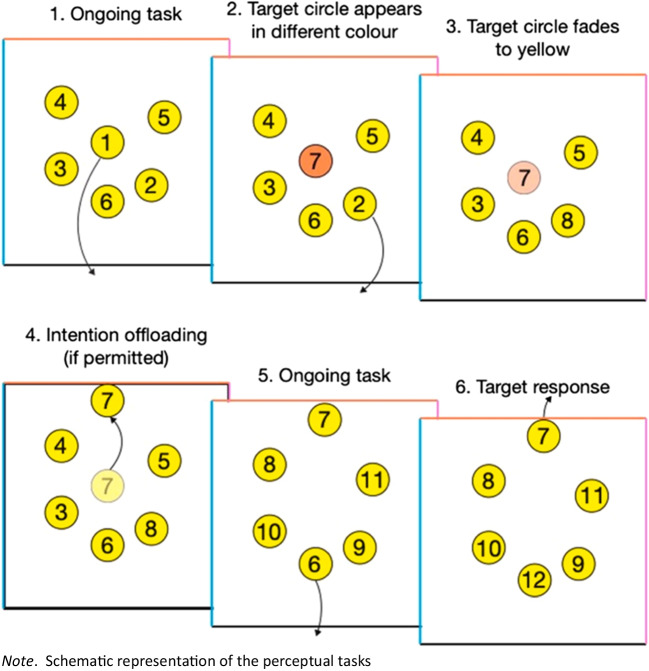
Schematic representation of the intention offloading task where participants have to drag the circles to the bottom of square in numerical order. Occasionally, new circles initially appear in blue, orange, or pink (rather than yellow), before fading to yellow matching the other circles

This task was similar to the one used in our previous study (see Sachdeva & Gilbert, 2020, no-reward group). As can be seen in Fig. 2, on each trial, participants were presented with six yellow circles randomly positioned within a square. Each circle contained a number, and using their mouse, participants had to sequentially (in numerical order) drag the circles to the bottom of the square. Each time a circle was dragged to the bottom of the square, a new circle appeared in its original location, continuing the numerical sequence. This continued until all 25 circles were dragged out of the square. Occasionally, new circles (described as special circles to the participant) initially appeared in blue, orange, or pink rather than yellow. These colors corresponded with the left, top, and right side of the square, respectively. Two seconds after appearing on the screen, their color faded to yellow matching the other circles. When a special circle appeared (e.g., in orange), it represented an instruction to the participant that it should eventually be dragged to its corresponding side of the square (e.g., to the top) when it was reached in the numerical sequence. For example, a participant first drags 1 to the bottom of the screen where it disappears. An orange 7 appears in its place, fading to yellow after 2 s. Meanwhile, the participant drags circles 2–6 to the bottom of the screen a before dragging 7 to the top. Therefore, special circle instructed participants to form a delayed intention to drag that circle to a nonstandard location when it was eventually reached in the sequence.

Importantly, these special circles could be remembered in two different ways. Participants could either rely on their own representation of where that circle needs to be dragged (i.e., no reminder). Or participants could set an external reminder as soon as that circle appeared on the screen by moving it near the location (e.g., top) where it eventually needed to be dragged. In this case there was no need to maintain an internal representation of the intended behavior as it was directly cued by the circle’s position.

One trial consisted of a numerical sequence from 1 to 25. Within this sequence a total of 10 target (special) circles appeared. These targets were allocated to 10 numbers between 7 and 25, spaced as evenly as possible. This meant that participants had to remember simultaneous intentions and it was unlikely that they would be able to remember all of them without setting external reminders. The target circles were randomly allocated to the left, top. and right positions of the square.

The main memory task consisted of a series of 17 experimental trials where each trial consisted of a full set of 25 circles and ten targets. On even-numbered trials, participants were forced to use either an internal strategy (unaided memory) or an external strategy (reminders). To force an internal strategy, all circles (apart from the next in sequence) were fixed on the screen and could not be moved, ensuring that participants could not set reminders for the target circles. To force an external strategy, participants were prevented from dragging circles out of the box until after they had adjusted the location of forthcoming target circles. On the remaining nine odd-numbered trials, they were asked to choose between the internal and external strategies before starting the trial. Prior to beginning a forced internal or external trial, participants were informed on which strategy they had to use. Participants were also told that they scored points every time they correctly dragged a target circle to its instructed location. On forced internal and external trials, each correct target response was worth 10 points. On choice trials, participants scored 10 points for each correct target response if they chose to use their own memory. If they chose to use reminders, they were offered a lower number of points (between 1 and 9) for each correct target response. After each trial, participants were told the number of points they had scored in the experiment so far. They were told to score as many points as possible, and that on choice trials they should choose whichever strategy they believed would allow them to score more points.

Unlike Sachdeva and Gilbert (2020), feedback was provided in the following form: when a target circle was correctly dragged to its corresponding side of the square box, it turned green before disappearing. When any circle was dragged to an incorrect target location (left, right, or top), it turned red before disappearing. When circles were dragged to the bottom of the box, they always turned purple before disappearing, so in this case no feedback was provided. Furthermore, while Sachdeva and Gilbert (2020) manipulated the difficulty of the practice trials, in this study practice trials were of the same difficulty as the experimental trials. That is, there were always ten targets on each trial.

In addition to the memory task, participants were asked for two confidence ratings using a continuous slider ranging from 0% (they thought that they would never respond correctly to any of the target circles) to 100% (they thought that they would respond correctly to all the target circles). One of these sliders was presented to them after they finished the practice trials (called the pre-task confidence judgment) and the other one was presented at the end of the memory task (called the post-task confidence judgment). A demonstration of the memory task can be viewed at: https://cognitiveoffloading.net/chhavi/offloadingDemo/WebTasks.html.

### Measures

The key dependent measures in the experiment were as follows:

- ***Perceptual tasks***
  **Mean accuracy in the Number task:** Measure of task performance (i.e., proportion of correct responses) in the number task.
  **Mean accuracy in the Contrast task:** Measure of task performance (i.e., proportion of correct responses) in the contrast task.
  **Mean efficiency in the Number task:** This measure reflects how well a participant can discriminate correct from incorrect responses in the number task. To do this, we used the *meta-d’/d’* ratio (*M*-ratio) to calculate metacognitive efficiency (Maniscalco & Lau, 2012). The *M*-ratio is a metacognitive measure that captures how well individuals’ confidence ratings align with their actual perceptual decision-making accuracy. A higher *M*-ratio reflects better alignment between confidence rating and actual performance, indicating high metacognitive efficiency. To calculate metacognitive efficiency we first discretized the trial-by-trial confidence ratings of each participant into six equal bins using quantile ranks and then we fit *meta-d’* to each participant’s confidence rating using the maximum likelihood estimation model implemented in MATLAB (version 2020a) (http://www.columbia.edu/~bsm2105/type2sdt/) by Maniscalco and Lau (2012).
  **Mean efficiency in the Contrast task:** This measure reflects how well a participant can discriminate correct from incorrect responses in the contrast task. This was calculated using the same method as metacognitive efficiency in the number task.
  **Confidence in the Number task: **Mean confidence across trials in the number task. This reflects each participant’s overall tendency to report high or low confidence irrespective of their performance.
  **Confidence in the Contrast task:** Mean confidence across trials in the contrast task. This reflects each participant’s overall tendency to report high or low confidence irrespective of their performance.
  **Post-task confidence rating in** **the Number task:** Additional measure of confidence from a single rating given by participants at the end of the number task reflecting the proportion of trials on which they thought they were able to correctly discriminate between the two stimuli if they were presented with more trials.
  **Post-task confidence rating in the Contrast task:** Additional measure of confidence from a single rating given by participants at the end of the contrast task reflecting the proportion of trials on which they thought they were able to correctly discriminate between the two stimuli if they were presented with more trials.
- ***Memory task***
  **Forced internal accuracy (ACC**_**FI**_**):** Mean target accuracy (i.e., proportion of target circles correctly dragged to the instructed location) on forced internal trials.
  **Forced external accuracy (ACC**_**FE**_**):** Mean target accuracy (i.e., proportion of target circles correctly dragged to the instructed location) on forced external trials.
  **Optimal indifference point (OIP):** Target value offered with reminders at which an unbiased individual should be indifferent between the two options. This value was based on the ACC_FI_ and ACC_FE_. As in Gilbert et al. (2020), the OIP can be calculated as follows:
  $$\mathrm{OIP }= (10\times {{\text{ACC}}}_{{\text{FI}}})/{{\text{ACC}}}_{{\text{FE}}}.$$
  In a departure from our pre-registered analysis plan, for each participant the ACC_FI_ measure was derived from just two of the four forced internal trials, randomly selected. The reason for this was to ensure that independent data were entered into correlational analyses comparing the OIP with another measure which was also derived from ACC_FI_ (see below).
  **Actual indifference point (AIP):** Estimated point at which participants were actually indifferent between the two strategy options. As in Gilbert et al. (2020), this was calculated by fitting a sigmoid curve to the strategy choices (0 = own memory; 1 = reminders) across the nine target values (1–9) using the R package “quickpsy” bounded to the range 1–9. The AIP can be seen as an index of offloading frequency (similar to the externalizing proportion in Gilbert, 2015b). A low AIP indicates that the participant only required a small target value to choose external reminders and so, they set reminders on a larger number of trials. A high AIP indicates that they rarely chose external reminders requiring a high target value to choose to offload.
  **Reminder bias:** Defined as OIP-AIP, which would yield a positive number for bias towards using more reminders than would be optimal.
  **Confidence (pre-task):** Response made on the pre-task confidence scale. This is a single-point estimate of confidence given by participants before beginning the main experimental trials.
  **Metacognitive bias (pre-task):** Difference between the pre-task confidence rating and actual accuracy on forced internal trials. A positive number would indicate overconfidence and a negative number would indicate underconfidence. In a departure from the pre-registered analysis plan, this was calculated from the two remaining ACC_FI_ trials that were not used to calculate the OIP, to ensure that the correlation between OIP and metacognitive bias was based on independent data.
  **Confidence (post-task):** Response made on the post-task confidence scale. This is a single-point estimate of confidence given by participants at the end of the experiment.
  **Metacognitive bias (post-task):** Difference between the post-task confidence rating and actual forced internal accuracy. In a departure from the pre-registered analysis plan, this was calculated from the same two ACC_FI_ trials as the pre-task metacognitive bias measure.

### Exclusion criteria

#### Memory task

In the memory task, participants were excluded if they satisfied any of the following pre-registered criteria: accuracy in the forced internal lower than 10%, accuracy in the forced external condition lower than 70%, accuracy in the forced internal trials higher than accuracy in the forced external trials as this would imply that reminders did not improve performance making data uninterpretable (*n* = 4), a negative correlation between target value and the likelihood of choosing to use reminders, which would suggest random or counter-rational strategy choice behavior (*n* = 2). Participants were also excluded if their reminder bias exceeded 3 median absolute deviation (MAD) units (Leys et al., 2013) (*n* = 1) or if their metacognitive bias exceeded 3 MAD units.

#### Perceptual tasks

In the perceptual tasks, participants were excluded if their average accuracy across Number and Contrast discrimination tasks exceeded 3 MAD units as this would suggest a failure of the staircase procedure and/or frequent guessing or random responses (*n* = 4).

### Procedure

Before commencing the study, all participants provided informed consent. Once completed, they proceeded to begin either the perceptual discrimination tasks first and the intention offloading task second, or vice versa.

#### Perceptual tasks

The two perceptual tasks were presented in randomized order. An example stimulus with minimum difficulty was presented for 1,000 ms and participants were asked to make a response. If an incorrect response was given, another example stimulus was presented. Once participants made a correct response, they were presented with five more trials where stimuli were presented for 800 ms. Participants needed to respond correctly to all stimuli otherwise they were asked to repeat these trials. Once five correct responses had been made, participants were presented with five more trials with stimuli presented for 250 ms. They needed to respond correctly to at least four of these stimuli before being able to continue the experiment.

Participants were then presented with 40 practice trials and from this point onwards difficulty was adjusted with a two-down-one-up staircase procedure where task difficulty increased by one step after two consecutive correct responses were made and decreased by one step after one incorrect response. After these practice trials, participants were introduced to the trial-by-trial confidence scale. Instructions for this were as follows: “Now that you have had some practice with this task, we would like to introduce you to another element. After each discrimination judgment, you will be asked to give a rating of how confident you are that you responded correctly. You will be presented with a scale ranging from 50% to 100%, where 50% means that your response was a complete guess and 100% means that you are absolutely certain that you answered correctly. To give your rating, you can click anywhere on the blue slider.” Participants then performed a further ten trials with the confidence judgment scale.

They then performed two blocks of 75 main trials where they were presented with the confidence judgment scale after each perceptual discrimination response. Finally, participants were asked to give their post-task confidence rating with the following instructions: “Now that you have finished this task, we would like you to tell us how accurately you think you can perform the task if there were more trials. Please use the scale below to indicate the percentage of times you can correctly discriminate between the two patterns, on average; 100% would mean that you can always get every single one correct. 50% would mean that every response was a complete guess, like tossing a coin for each answer.”

After completing the post-task confidence rating, the other perceptual task (Number or Contrast) was administered in an identical manner.

#### Memory task

Participants were first presented with seven circles without any targets so that they could practice simply dragging the circles to the bottom of the screen. Next, instructions for how to respond to targets was presented and participants performed one practice trial involving eight circles and one target. They were only allowed to continue to the next phase of the task if they responded correctly to the target, otherwise they were asked to repeat the trial. Following this, participants received two practice trials with 25 circles and ten targets. After these two practice trials, participants were asked to give their pre-task confidence rating with the following instructions: “Now that you have had some practice with this task, we would like you to tell us how accurately you think you can perform the task. Please use the scale below to indicate the percentage of special circles you can correctly drag to the instructed side of the square, on average; 100% would mean that you can always get every single one correct. 0% would mean that you can never get any of them correct.” After giving their response on the pre-task confidence scale, participants were presented with one practice trial which instructed them on how to set reminders by dragging target circles next to their intended side of the square (Gilbert et al., 2020, Experiment 3; Sachdeva & Gilbert, 2020).

After completing the practice trials, participants were introduced to the forced internal, forced external and choice conditions. They were also informed on how the points system worked in the task (see Gilbert et al., 2020; Sachdeva & Gilbert, 2020).

Once participants were familiarized with how the task worked, they were presented with the 17 main experimental trials as described in the design section. After each trial, they were able to see the total number of points they had scored since the start of the experimental trials.

After finishing the main experimental trials, participants were presented with the post-task confidence scale as follows: “Now that you have finished this task, we would like you to tell us how accurately you think you can perform the task without any reminders if there were more trials. Please use the scale below to indicate the percentage of the special circles you can correctly drag to the instructed side of the square, on average; 100% would mean that you can always get every single one correct. 0% would mean that you can never get any of them correct.”

Once participants completed both tasks, they were thanked, debriefed, and paid for their time.

## Results

We followed our pre-registered analysis plan with the following exception. The original pre-registration stated that “All statistical tests will be one-tailed”; however, we realized that this was not described with sufficient clarity, including some cases where the predicted direction of the effect was not clearly specified. We therefore took the conservative approach of reporting two-tailed tests throughout with the exception of the correlations between reminder bias in the memory task and confidence in the perceptual tasks. This was clearly specified in the pre-registration as a one-tailed test with a specified direction (see page 3 of pre-registration) and it was the basis of the power calculation for determining the sample size. We would also like to note that powering the experiment for a one-tailed test, reduces the power for the two-tailed analyses that are reported. Therefore, these analyses should be considered exploratory. All analyses were conducted using R (version 4.0.3).

### Memory task

See Table 1 for a summary of results from the intention offloading task. First participants’ metacognitive bias scores were investigated. This was the difference between their responses on the two confidence scales and their accuracy on the forced internal trials. One-sample *t*-tests (compared to zero) showed that participants were significantly underconfident when they made their first confidence judgment (*t*(137) = 7.61, *p* < .001, *d* = .65) and when they made their second confidence judgment (*t*(137) = 1.99, *p* = .049, *d* = .17).

**Table 1:** Means and standard deviations of behavioral results from the Memory task

	Mean	Standard deviation
Forced external accuracy (%)	98.59	2.43
Forced internal accuracy (%)	62.34	16.81
Confidence rating (pre-task)	44.19	23.92
Confidence rating (post-task)	58.86	23.86
Metacognitive bias (pre-task)	-18.27	28.19
Metacognitive bias (post-task)	-3.61	21.33
OIP	6.31	1.79
AIP	3.94	2.46
Reminder bias	2.37	2.28

Table showing means and standard deviations of behavioral results from the memory task. OIP = optimal indifference point; AIP = actual indifference point

Then participants’ reminder bias was investigated using a one-sample *t*-test (compared to zero). It was found that participants were significantly biased towards using reminders (*t*(137) = 12.18, *p* < .001, *d* = 1.04).

A paired-samples *t*-test between the pre-task and post-task metacognitive bias scores was conducted to investigate whether participants’ metacognitive bias changed between the two ratings. Participants (although still underconfident) were significantly less underconfident in their post-task metacognitive bias ratings (*t*(137) = 6.37, *p* < .001, *d* = .54).

**Fig. 3:**
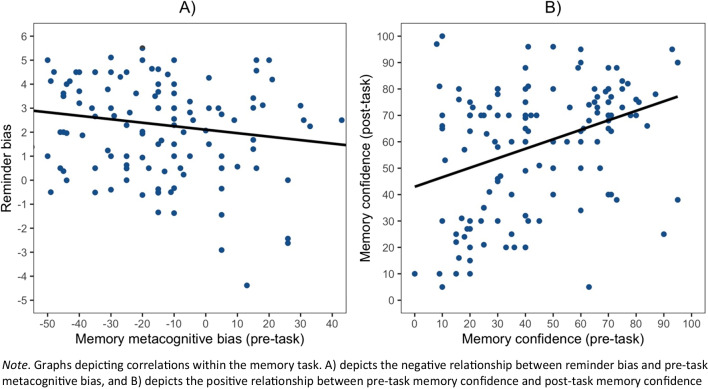
*Correlations within the Memory task*

To investigate the relationship between reminder bias and pre-task metacognitive bias, a Pearson’s correlation was conducted. A significant negative correlation (*r*(136) = -.17, *p* = .036) between these measures was found: participants who were more underconfident in their pre-task confidence ratings displayed a higher bias towards using reminders (see Fig. 3A). A similar negative correlation was found between participants’ reminder bias and their pre-task confidence rating (i.e., their raw confidence rather than under-/overconfidence as measured by metacognitive bias) (*r*(136) = -.21, *p* = .01).

Finally, to investigate the relationship between the two metacognitive confidence ratings (pre-task and post-task), a Pearson’s correlation was conducted. There was a significant positive correlation between the two confidence ratings (*r*(136) = .36, *p* < .001) where participants who had lower confidence in their pre-task ratings also had lower confidence in their post-task ratings (see Fig. 3B).

### Perceptual tasks

See Table 2 for a summary of results. Average accuracy in the perceptual tasks was at around 69.6%, indicating that the staircase procedure worked at maintaining participants’ accuracy approximately mid-way between chance and ceiling levels.

**Table 2:** Table showing the behavioral means and standard deviations from the perceptual task

	Number task		Contrast task	
	Mean	Standard deviation	Mean	Standard deviation
Accuracy (%)	71.13	2.2	68.05	2.32
Metacognitive efficiency	0.57	0.36	0.67	0.42
Confidence	73.09	9.05	74.82	9.63
Confidence rating (post-task)	69.7	9.65	70.46	11.18

A paired-samples *t*-test showed that accuracy in the Number task was higher than accuracy in the Contrast task (*t*(137) = 10.75, *p* < .001, *d* = .91). However, mean confidence rating (*t*(137) = 3.63, *p* < .001, *d* = .31) and metacognitive efficiency (*t*(137) = 2.38, *p* = .02, *d* = .20) were higher in the Contrast task than in the Number task. Post-task confidence ratings did not differ between the two tasks (*t*(137) = .88, *p* = .38, *d* = .07).

**Fig. 4:**
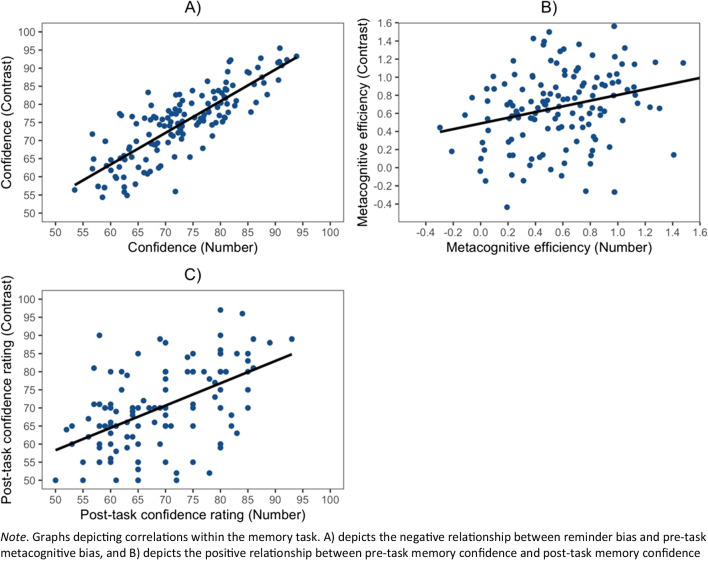
*Correlations within the Perceptual tasks*

Pearson’s correlations showed that apart from accuracy (r(136) = -.10, p = .23), every other measure in the perceptual tasks correlated significantly with their analogous measure in the other task, demonstrating that they capture meaningful variance (mean confidence: *r*(136) = .82, *p* < .001 A); metacognitive efficiency: *r*(136) = .27, *p* = .001 (Fig. 4B); and post-task confidence rating: *r*(136) = .53, *p* < .001 (Fig. 4C)). Furthermore, mean confidence and the single post-task confidence rating at the end of each task were significantly intercorrelated where participants who displayed lower confidence in their mean trial-by-trial ratings also gave lower confidence on the post-task confidence scales (*rs*(136) > .45, *ps* < .001).

Although metacognitive efficiency in the Contrast task was only correlated with its analogous measure in the Number task, metacognitive efficiency in the Number task was also correlated with mean confidence in both the Number task (*r*(136) = -.17, *p* = .04) and the Contrast task (*r*(136) = -.18, *p* = .03).

### Intercorrelations between perceptual and offloading tasks

**Fig. 5:**
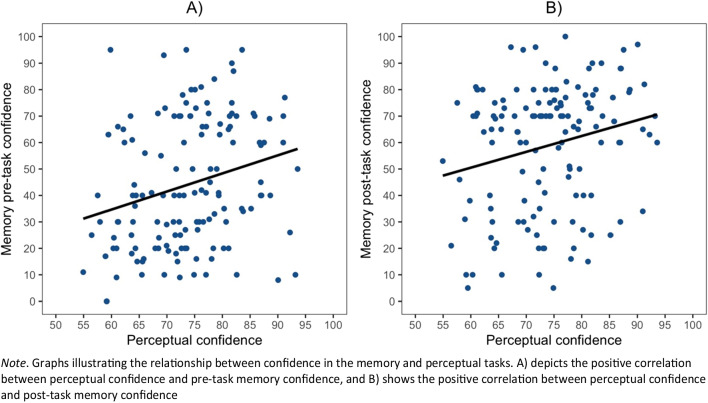
*Relationship between Memory and Perceptual Confidence*

To investigate the relationship between measures from the perceptual tasks and the memory task, the measures of accuracy, mean confidence, metacognitive efficiency and post-task confidence ratings were collapsed across the two perceptual tasks. To investigate our predictions on the cross-domain metacognitive signals between the perceptual and memory tasks, Pearson’s correlations were conducted on the collapsed scores detailed above and those derived from the memory task. We found that mean perceptual confidence was significantly correlated with pre-task (*r*(136) = .25, *p* = .002) and post-task (*r*(136) = .22, *p* = .008) memory confidence where participants who displayed higher confidence in their perceptual performance also displayed higher confidence in their memory performance (see Fig. 5A and Fig. 5B, respectively). Although there was a trend towards a negative correlation between mean perceptual confidence and reminder bias (*r*(136) = -.14, *p* = .057, one-tailed as specified in the pre-registration), it did not pass the conventional threshold for statistical significance. There was also no significant correlation between post-task perceptual confidence and reminder bias (*r*(136) = -.08, *p* = .17).

**Fig. 6:**
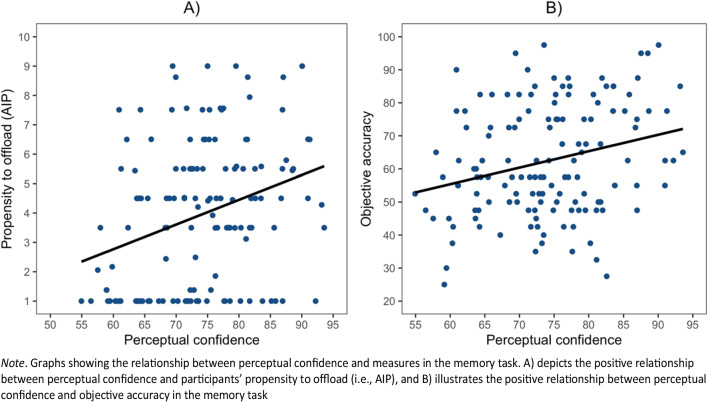
*Relationship between Perceptual Confidence and Measures in the Memory task*

Interestingly, both mean perceptual confidence (*r*(136) = .30, *p* < .001) (see Fig. 6A) and post-task perceptual confidence ratings (*r*(136) = .28, *p* < .001) were positively correlated with AIP, which is an inverse measure of how often participants choose to set reminders in the memory task. This shows that participants with higher confidence in their perceptual judgments not only tended to predict better performance in the memory task, but also set fewer reminders in the memory task. Furthermore, mean perceptual confidence (*r*(136) = .26, *p* = .002) (see Fig. 6B) and post-task perceptual confidence (*r*(136) = .35, *p* < .001) were significantly correlated with forced internal accuracy in the intention offloading task. So even though perceptual confidence was not correlated with perceptual accuracy (which was stabilized using a staircase procedure), participants with higher confidence in their perceptual confidence judgments also had greater accuracy in the memory task

As per the pre-registration, the correlation coefficient derived from the association between mean perceptual confidence and reminder bias, and the correlation coefficient derived from the association between pre-task memory confidence and reminder bias, were then compared to investigate whether one was significantly more predictive of reminder bias than the other. The difference between these two correlation coefficients was not significant (*z* = -.69, *p* = .49).

Since a significant correlation between perceptual confidence and AIP was found, an additional analysis that was not pre-registered was conducted. This analysis compared the correlation coefficient derived from the correlation between mean perceptual confidence and AIP and the correlation coefficient derived from the correlation between pre-task memory confidence and AIP. A significant difference between these two correlation coefficients was not found (*z* = -.10, *p* = .92). Therefore, there was no evidence for any contribution of task-specific metacognitive signals over and above domain-general ones.

An additional analysis comparing the correlation between perceptual confidence and AIP and perceptual confidence and reminder bias was also conducted. This was significant (*z* = -2.88, *p* = .004), adding further support for a greater influence of domain-general metacognitive signals on AIP (i.e., propensity to set reminders) than reminder bias (i.e., preference for reminders, relative to the optimal strategy).

As predicted, metacognitive efficiency in the perceptual tasks did not correlate with any of the measures derived from the memory task (*rs* < .13, *ps* > .14).

### Domain-general versus domain-specific signals

To investigate whether reminder bias was influenced by both domain-general and domain-specific confidence signals, a multiple linear regression with reminder bias as the dependent variable and perceptual confidence and pre-task memory confidence as independent variables was conducted. The perceptual confidence measure was generated by transforming the mean trial-by-trial confidence and post-task confidence ratings into *Z* scores. These scores were then collapsed across the two measures. There was a significant effect of pre-task memory confidence (*β* = -.02, *SE* = .008, *t*(135) = -2.18, *p* = .03) but not perceptual confidence (*β* = -.18, *SE* = .22, *t*(135) = -.80, *p* = .43). This shows that pre-task memory confidence accounts for significant variance in reminder bias, in addition to variance attributable to perceptual confidence. Therefore, reminder bias is likely influenced by domain-specific metacognitive signals.

Since there was a significant positive correlation between perceptual confidence and AIP, an additional multiple linear regression that was not included in the pre-registration was conducted. In this model, AIP was included as the dependent variable and pre-task memory confidence rating and perceptual confidence were included as independent variables. This analysis evaluated whether the use of reminders was related to domain- specific along with the domain-general confidence found above. In this model, both pre-task memory confidence rating (*β* = .03, *SE* = .008, *t*(135) = 3.02, *p* = .003) and perceptual confidence (*β* = .66, *SE* = .23, *t*(135) = 2.94, *p* = .004) were significant, suggesting that AIP was influenced by both domain-general and domain-specific confidence signals.

## Discussion

The main aim of the current study was to examine the link between reminder-setting behavior in a memory task and the domain-general versus task-specific confidence signals influencing this behavior. With regards to the memory task, the findings of previous research were replicated where it was found that decisions of whether or not to set reminders are linked to participants’ metacognitive evaluations (see Gilbert et al., 2020; Kirk et al., 2021). Therefore, confidence signals play an important role in cognitive offloading.

With regards to the perceptual tasks, it was found that metacognitive efficiency and confidence in one perceptual task was related to its analogous measure in the second perceptual task. These findings support those of Gilbert (2015b Experiment 2) and Song et al. (2011) who found that: (1) metacognitive sensitivity correlates with its corresponding measure in perceptual tasks, (2) confidence correlates with its corresponding measure in the perceptual tasks, and (3) metacognitive sensitivity and bias are not correlated with each other in perceptual tasks.

### Domain-general account of metacognitive confidence

With regards to the cross-domain associations, evidence for a domain-general component of metacognitive confidence was found where confidence in perceptual tasks was associated with memory confidence even though perceptual confidence was decorrelated from perceptual accuracy using a staircase procedure (see Gilbert, 2015b, Experiment 2; Mazancieux et al., 2020; McCurdy et al., 2013). This supports the domain-general account of metacognition that proposes that individuals use domain-general metacognitive signals when evaluating their performance (de Gardelle & Mamassian, 2014; Faivre et al., 2017). Furthermore, there was also a cross-domain link between perceptual confidence and reminder-setting in the memory task (i.e., AIP), where participants with lower perceptual confidence tended to set more reminders in the memory task.

The main novelty of this study was that in addition to participants’ overall level of reminder-setting behavior, their bias towards/away from setting reminders relative to optimal strategy was also investigated. As in earlier studies (see Gilbert et al., 2020; Kirk et al., 2021), both these measures were associated with memory confidence. However, unlike the overall index of reminder-setting behavior (i.e., AIP, *propensity* to offload), there was no significant correlation between perceptual confidence and reminder bias (i.e., *preference* to offload). Furthermore, the correlation between perceptual confidence and AIP was significantly greater than the correlation between perceptual confidence and reminder bias, substantiating the cross-domain confidence effects on AIP, but not reminder bias.

This finding could be explained by some of the results in the current study. Before doing this, it should be pointed out that the correlation between perceptual confidence and reminder bias was marginally significant and that it is difficult to interpret null results. However, one possible explanation for this result could be that even though perceptual confidence was dissociated from perceptual accuracy, it was still correlated with objective accuracy in the memory task. This suggests that perceptual confidence cannot be considered a “pure” measure of metacognitive bias as it relates to cognitive ability in some way. Thinking of metacognition as an inferential process rather than simply a read-out of cognitive performance (see Koriat, 2007, for a review), this could be considered a rational strategy. Since performance does tend to correlate across tasks (the *g* factor), on average, it is rational for an individual whose performance is high in one domain to predict higher performance in another domain. So, an individual with relatively good memory performance might also predict relatively good perceptual performance, even though the latter was stabilized by the staircase procedure. This could explain the link between perceptual confidence and memory accuracy.

One consequence of this link is that it would eliminate the correlation between perceptual confidence and reminder bias as people with low confidence in their perceptual judgments might tend to set more reminders in a memory task simply because they also *need* more reminders (since low perceptual confidence is linked to low memory performance). Therefore, the cross-domain link between perceptual confidence and reminder bias is weaker than the link between perceptual confidence and propensity to use reminders.

Another possible explanation for this comes from the results of Ball et al. (2021). They found a similar result where in their study they used three different versions of the same memory task and found that even though the paradigm was the same, reminder bias only correlated with memory confidence in its own task but not with memory confidence from a different task. This result along with those presented in this study indicate that reminder bias is influenced by task-specific confidence signals.

It should also be noted that the confidence rating used in the perceptual tasks was a retrospective metacognitive judgment, while the pre-task memory confidence was a prospective metacognitive judgment. This dissimilarity between the two metacognitive measures could potentially account for the observed correlation between pre-task memory confidence and reminder bias, and not between perceptual confidence and reminder bias. To address this divergence in metacognitive assessments, future research should distinguish prospective from retrospective metacognitive judgments.

Furthermore, considering the notion of global metacognition (e.g., Rouault & Fleming, 2020) within the framework of intention offloading could be useful. Using structural equation modelling (SEM), future research could explore the presence of a global factor influencing metacognitive assessments across both perceptual and memory tasks, and the subsequent predictive relationship of this factor with performance in memory tasks.

### Metacognitive efficiency

The results presented in this study support the domain-specific account of metacognitive efficiency where it correlated with its analogous measure in the perceptual tasks but did not correlate with any of the measures in the memory task. This finding supports that of previous research where the majority of results have concluded in favor of a domain-specific account for metacognitive efficiency (Baird et al., 2013, 2015; Gilbert, 2015b, Experiment 2; Morales et al., 2018). This is also corroborated by neurophysiological results where perceptual metacognitive sensitivity has been found to be related to the anterior prefrontal cortex (aPFC) (Baird et al., 2013, 2015; McCurdy et al., 2013) and lesions to the aPFC have been shown to selectively impair perceptual sensitivity while leaving memory task performance intact.

In this study, although metacognitive efficiency and confidence were correlated with their analogous measures in the two perceptual tasks, metacognitive efficiency in the Number task was also associated with confidence in the Number task and the Contrast task. This finding is surprising as these two measures are thought to be independent of each other (see Galvin et al., 2003; Song et al., 2011). However, these results support those of Shekhar and Rahnev (2020), who found that metacognitive efficiency *depended* on confidence level, where metacognitive efficiency becomes less reliable for higher confidence criteria. So, in some samples, it is possible to find a correlation between metacognitive efficiency and metacognitive confidence (Shekhar & Rahnev, 2020).

### Implications

Metacognition, the ability to monitor mental processes, serves as the theoretical foundation of our experiment. Drawing on the conceptual framework in metacognition research (for a review, see Mazancieux et al., 2023), we navigated the distinction between domain-general and domain-specific metacognitive processes in cognitive offloading. This distinction is crucial in understanding whether individuals leverage shared metacognitive signals or distinct resources when evaluating their performance across diverse tasks.

The theoretical implications from this study provide insights into the architecture of metacognition and its effects in real-world decision-making. First, Mazancieux et al. (2023) distinguish between decisional and adecisional forms of metacognition, where decisional metacognition involves metacognitive evaluations based on specific decisions made during the task, while adecisional metacognition includes broader beliefs and knowledge about functioning that are not directly tied to specific first-order decisions. The observed correlation between perceptual confidence and propensity to offload in this study suggests the presence of decisional metacognition. As noted by Mazancieux et al. (2023), studies consistently find that metacognitive bias is stable across various domains, including perception and memory. However, this pattern appears to be more complex for metacognitive efficiency. While there are some studies that report positive correlations across memory and perception tasks (e.g., Lee et al., 2018; Lund et al., 2023; Mazancieux et al., 2020; Morales et al., 2018), there is variability in the magnitude of these correlations. Mazancieux et al. (2023) also note that correlations across domains in metacognitive efficiency might just reflect the influence of domain-general metacognitive bias, where in practice correlations between metacognitive efficiency and bias are found (Xue et al., 2021). Therefore, as metacognitive bias is mainly domain-general (Lee et al., 2018; Lund et al., 2023; Mazancieux et al., 2020), a spurious domain-general metacognitive efficiency could arise when bias and efficiency correlate. Future research should disentangle the two where the contribution of both can be investigated across domains.

## Conclusion

In conclusion, the results of this study found evidence for the influence of both domain-general and task-specific confidence signals on reminder-setting behavior. However, these domain-general signals did not fully explain metacognitive influence on reminder-setting behavior especially with respect to reminder bias that did not correlate with confidence across domains. There was also no evidence for a link between perceptual metacognitive efficiency and reminder-setting behavior. These results suggest that although metacognitive interventions could have some cross-domain influence on offloading behavior, task- specific interventions are more likely to have a stronger impact, especially when it comes to preference to offload.

## Data Availability

The data that support the findings of this study are openly available via the Open Science Framework (OSF) at: https://osf.io/9efjb/.

## Code availability

All statistical analyses were coded using R (version 4.0.3). Code used for analysis is openly available via the OSF at https://osf.io/9efjb/.
